# Segregation Analysis Suggests That a Genetic Reason May Contribute to “the Dress” Colour Perception

**DOI:** 10.1371/journal.pone.0165095

**Published:** 2016-10-21

**Authors:** Feifei Xiao, Guoshuai Cai, Heping Zhang

**Affiliations:** 1 Department of Epidemiology and Biostatistics, University of South Carolina, Columbia, SC, 29208, United States of America; 2 Department of Genetics, Geisel School of Medicine at Dartmouth, Hanover, NH, 03755, United States of America; 3 Department of Biostatistics, Yale University School of Public Health, New Haven, CT, 06520, United States of America; Johns Hopkins University Bloomberg School of Public Health, UNITED STATES

## Abstract

In early 2015, the debate of blue-black and white-gold color perception from “the dress” became an overnight internet phenomenon. According to the vote from the online social network Twitter, more people observed white-gold colors than those who observed blue-black colors. Biological explanations have been proposed by neurologist and other scientists, most of which mainly focus on the bias of color perception from visual cortex assuming different illuminants as backgrounds. The goal of this study was to investigate the genetic reason that might be underlying this phenomenon. We carried out a preliminary survey study using four complex pedigrees and examined the inheritance mode influencing the ability to perceive the real colors, blue-black, from the photograph. We evaluated the likelihood of sporadic, major gene in Mendelian mode, major gene in non-Mendelian mode and environmental models. Complex segregation analyses indicated that the inheritance was probably due to a non-Mendelian major gene effect. Our study also indicated the importance of environmental or epigenetic factors in this color perception trait.

## Introduction

On February 27, 2015, a Tumblr user "swiked" posted a washed-out photograph of a dress and proposed a question: "Guys please help me: is this dress white and gold, or blue and black?" However, this simple, but peculiar question quickly spread throughout the internet and triggered intense debates globally, as so called “the dress” phenomenon. Immediately, a poll was conducted by the online social network Twitter about what colors people observe from the photograph. According to the results from the poll, more people believed it was a white-gold (WG) dress in the dark shadow. Fewer people perceived it as a blue-black (BB) dress, though washed out by bright light in the room where the photograph was taken. A Photoshop analysis showed the dress’ pixels were actual brown and blue, colors associated with natural illuminants [1]. Subsequently, this prompted an immediate online discussion of the underlying mechanisms of color perception.

Generally, the perception of color is achieved by a complex process that starts with the retina in human eyes [2]. When an object is exposed to light, the light reflection of the object enters the eyes through the lens, hits the retina in the back of eye and the signals are transmitted to the visual cortex as part of the brain through neural connections; the brain then processes these signals into an image [3]. Usually, the human brain is essentially powerful enough to extract the real color of the object among noises. However in “the dress” phenomenon case, the dress was overexposed under a yellow light and the background in the room was obscure, causing variation for color extraction among human individuals [4]. In other words, the variation in the dress color perception was due to variation of color perception in that particular reflected light, rather than perception of the real colors. Beyond the biological explanations that have been offered, it is intriguing to investigate whether this type of variation is caused by genetic factors.

Evidence has suggested that many defects in human color perception are influenced by genetic factors. Color blindness, such as red-green color blindness, is a well-studied individual variation that is a sex chromosome linked phenotype [5]. The predisposing gene is located on the sex chromosome, X, therefore resulting in higher prevalence in men compared with women. Blue color blindness or tritanopia is another rare color defect due to a single locus mutation on chromosome 7 [6]. Although the color perception discrepancy in the dress case may not cause significant impact on human living, it is still inspiring to understand the possible underlying genetic architecture and biological pathways, which might provide novel insight into the understanding of the human complicated optical system.

To test whether “the dress” phenomenon is similar to the red/green color blindness and tritanopia as a genetic trait, we carried out a preliminary survey study and performed segregation analyses to test the transmission mode of perceiving the real colors, blue-black, in the dress photograph. Complex segregation analysis was performed to reveal the presumed modes of inheritance using four unrelated families. Our results indicated the inheritance was probably due to a non-Mendelian major gene effect. The importance of environmental or epigenetic factors in this color perception trait is also indicated in this study.

## Methods

### Data collection

**Fig 1:**
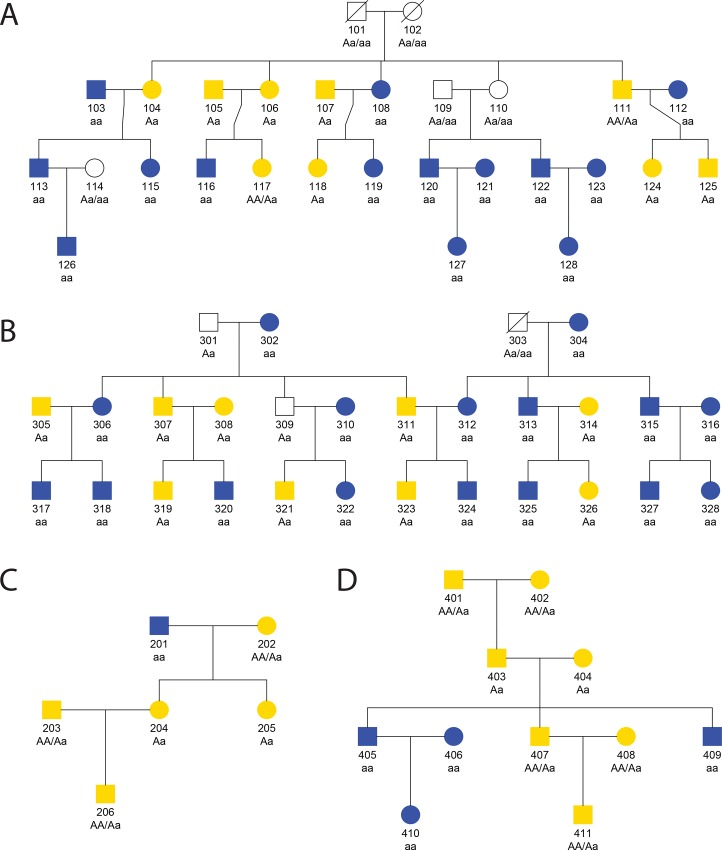
The pedigree tree for all four pedigrees. (A) family 1; (B) family 3; (C) family 2 and (D) family 4. The numeric number denotes the individual IDs. The circles denote females and the squares denote males. Individuals who observed blue and black were labeled in blue; individuals who observed white and gold were labeled in yellow. White color denotes missing data. The slash denotes decease. *AA*, *Aa* or *aa* was hypothesized genotypes under the assumption that the ability of observing blue-black colors from the dress picture was controlled by an autosomal locus with alleles *A* and *a* where *a* was the recessive allele.

Four Han Chinese families including 70 subjects in total were collected in the survey study. The probands in the four families were subjects with IDs 115, 201, 324 and 409 (Fig 1), respectively. Probands in our study were defined as the first family member who observed the BB colors from the dress photograph and attracted our attention to study the whole family. Then the probands displayed the same photograph to their family members in the pedigree, and tested their color perception independently. Basic demographic information was collected from each subject, such as gender and age. Each subject was asked to answer two questions: (1) Do you have blue color blindness? (2) What color is this dress, blue and black, white and gold or other? Subjects with blue color blindness would be exempt from the second question and excluded from our study. As a result, among the four families, no one reported blue color blindness. To validate the color perception from the dress photograph, all subjects took a second round test for the second question on the following day. Subjects who switched their answer in the color perception were recorded. Those who observed blue and brown were classified as BB as the perception of brown can be explained as the washed-out color of black under the yellow light. All subjects were then classified into three groups, BB, WG and switch, according to what colors they observed in the photograph. BBs were subjects who observed dark colors whereas WGs were those who observed light colors from the photograph. Summary of the color perception of the four pedigrees is shown in Table 1.

**Table 1:** Summary of the four pedigrees.

Phenotype	Family 1		Family 2		Family 3		Family 4		Total
	Male	Female	Male	Female	Male	Female	Male	Female	
**BB**	6	8	1	0	8	8	2	2	35
**WG**	4	5	2	3	6	3	4	3	30
**N/A**	1	2	0	0	2	0	0	0	5
**Total**	11	15	3	3	16	11	6	5	70

BB: blue-black color perception; WG: white- gold color perception; N/A: missing information.

The primary tool that we used to distribute and display the photograph was the cell phone through the social media software WeChat. We required the probands to use transparent screen color when the photograph was displayed on the cell phone during the tests. The dress photograph was originally downloaded from Wikipedia with link as follows: https://en.wikipedia.org/wiki/The_dress_%28viral_phenomenon%29#/media/File:The_Dress_%28viral_phenomenon%29.png. The saved photograph was then used as the primary source to test color perception in the survey.

This survey study was carried out in accordance with approved guidelines, and all protocols and consent procedures were approved by the Yale University Human Subjects Committee. This research was exempt from IRB review and written informed consent by the committee. Therefore, verbal informed consent was obtained from all participants, and then recorded and documented in the Department of Biostatistics, Yale School of Public Health. The verbal informed consent of children less than 16 years old was obtained from the guardians.

### Statistical analysis

Excluding subjects with missing information, all other participants were classified by gender (male or female) for BBs and WGs separately. Fisher’s exact test was performed to test the gender discrepancy in the dress photograph color perception using the 2x2 table. Then, to test the inheritance pattern of perceiving BB or WG colors from the dress photograph, we extracted all nuclear families embedded in the four complex pedigrees. In total, 21 nuclear families were extracted and analyzed. Two transmission modes were considered for this color perception phenomenon, (i) a major autosomal gene controlled transmission and (ii) independent transmission. For each inheritance mode, χ^2^ test was performed to determine whether there was significant difference between the expected frequencies under the hypothesized inheritance mode and the observed frequencies of color perception phenotypes in different parental combinations of genotype or phenotype. To correct for ascertainment biases arising from non-random sampling as we selected four pedigrees with probands having BB color perception, we also performed the χ^2^ tests in the four pedigrees omitting the probands. P values < 0.05 were considered statistically significant in these tests.

Under the hypothesis of inheritance mode (i), a major autosomal gene controls the color perception of WG from the photograph. Segregation was assumed to be through a single autosomal locus with two alleles, *a* and *A*, allele *a* being the recessive trait allele. Accordingly, individuals with genotype *AA/Aa* were assigned to WGs and those with genotype *aa* were assigned to BBs for the parents in the nuclear families. Based on the parental genotypes, the expected frequencies of WGs (with genotypes *AA* or *Aa*) or BBs (with genotypes *aa*) in the offspring were calculated based on the estimated frequency of allele *a* in the population, which was inferred from the Twitter poll. The observed offspring phenotypes (WGs or BBs) were summarized in different parental combinations of genotype according to their color perception testing results. The degree of freedom for the χ^2^ test was the number of parental combinations minus one.

Under the hypothesis of inheritance mode (ii), no gene was responsible for the color perception from the photograph. Therefore, in the nuclear families, the expected frequency of BBs in the offspring should follow the frequency of BBs in the population, which was 0.32 according to the Twitter poll. For each parental combination of phenotype (BB-BB, WG-BB or WG-WG), we summarized the observed numbers of BBs and WGs in the offspring according to their color perception testing results. The degree of freedom for the χ^2^ test was the number of parental combinations minus one.

### Segregation analysis

To further explore the inheritance mode of the color perception of BB, we performed complex segregation analysis using the genetics software package S.A.G.E. [2012] Statistical Analysis for Genetic Epidemiology, Release 6.3: http://darwin.cwru.edu. For analysis of binary trait, we performed maximum likelihood method to estimate the parameters in each of the hypothesized mathematical models examined using the SEGREG program of SAGE.

For each model we examined, we assumed that the presence (or absence) of the putative trait allele influenced perception of BB from the dress photo. The regression multivariate logistic model for binary trait was then applied as suggested by Karunaratne and Elston [7]. As we mentioned in the previous section, in major gene models, segregation was assumed to be through a single autosomal locus with two alleles, *a* and *A*, allele *a* being the trait allele. Allele frequencies were denoted by q_a_ and (1- q_a_). The three genotypes would then be transmitted to the next generation according to Mendelian mode or non-Mendelian mode. The corresponding baselines for susceptibility were then β_aa_, β_Aa_ and β_AA_. Individuals with genotypes *aa*, *Aa* and *AA* are assumed to transmit allele *a* to their offspring with transmission probabilities τ_aa_, τ_Aa_ and τ_AA_.

In our analysis, we considered ten different models including sporadic models (without or with familial/residual association), Mendelian major gene models (dominant, recessive or additive), non-Mendelian major gene models (dominant, recessive or additive), environmental and tau AB free models. We also performed covariate analysis by including the presence of age or gender as a continuous or binary variable. In these models, parameters for susceptibility, genotype frequencies and transmission probabilities were fixed or freely estimated. The non-Mendelian major gene recessive model we mentioned here is in fact the transmission mode ii for chi-square test discussed in the previous section. The sporadic model we mentioned here is equivalent to the independent transmission mode, which assumed no familial association (FA). All other models were assuming equal parent-offspring and sibling-sibling correlations. In Mendelian major gene models, the transmission parameters were restrained to the Mendelian mode, where τ_aa_ = 1, τ_Aa_ = 0.5, and τ_AA_ = 0. In comparison, the transmission parameters were estimated from the data in the non-Mendelian major gene models. The environmental model assumed no gene transmission and the tao AB free model assumed fixed transmission for homozygotes with estimated heterozygotes transmission lying between. Moreover, Akaike Information Criterion (AIC) was used to select the most parsimonious one among these hypothesized models. The model with lower AIC value was considered to better fitting the data.

## Results

Among the four complex pedigrees, 70 subjects were tested for the color perception from “the dress” photograph, whereas 30 subjects reported to perceive WG colors, 35 subjects perceived BB colors, and 5 subjects had missing information. No one reported switch of color perception after the second round of test. We then studied the dichotomous trait of color perception of “the dress” in the photograph based on the testing records and biological information from the participants. Regarding the gender discrepancy in color perception (Table 1), we did not detect any significant difference between males and females using Fisher’s exact test (p-value = 0.89), suggesting that this trait was not sex chromosome linked.

Moreover, we assessed the family aggregation of the WG/BB phenotypes in the four pedigrees (pedigrees 1, 3, 2 and 4 are shown in Fig 1 in order). The 21 nuclear families were extracted from the four pedigrees for the following analyses. In pedigrees 1, 2, and 4, when both parents observed BB colors (such as parent pairs 120 and 121, 123 and 124, 315 and 316, 405 and 406), so did their offspring. Among pedigrees 1, 2, and 3, when parents observed BB and WG (such as parent pairs 103 and 104, 107 and 108, 111 and 112), the offspring presented mixed combinations of BBs and WGs. These preliminary findings motivated us to hypothesize that the ability to perceive BB from the photograph was controlled by a major gene with recessive trait allele. Specifically, people who observe BB are recessive homozygotes (*aa*) and those who observe WG are dominant homozygotes (*AA*) or heterozygotes (*Aa*). Under this assumption, the population frequency of phenotype BBs should be much less than that of the phenotype WGs in a random mating population. A poll conducted by the online social network Twitter showed that 32% of 3.4 million voters were BBs, therefore providing nontrivial support for this assumption [8].

Motivated by the preliminary findings, we further investigated the inheritance pattern of the dress color perception based on the four complex pedigrees. χ^2^ tests were performed to evaluate two possible inheritance modes, (i) a major autosomal gene controlled transmission and (ii) independent transmission, respectively. Inheritance mode i was proposed to demonstrate that the recessive homozygotes on a single locus would lead to color perception of BB. As a result, the *p*-value was 0.28 which failed to reject the inheritance mode of a major autosomal gene with a recessive trait allele controlled transmission (Table 2). Most of the statistical tests in the seven presumed parental genotype groups were not able to reject this hypothesis.

**Table 2:** Summary of χ^2^ test in nuclear families under the hypothesis of a single dominant gene controlled transmission.

Parent Geno.	Parent pairs	P_AA_ in Offs.	P_Aa_ in Offs.	P_aa_ in Off.	Obs. No.		Exp. No.		χ^2^	*P*-value
					WG	BB	WG	BB		
***Aa*, *aa***	(103, 104),(107, 108),(301, 302),(305, 306),(309, 310),(311, 312),(313, 314)	0	$\frac{1}{2}$	$\frac{1}{2}$	6	9	7.5	7.5	0.6	0.44
***Aa*, *Aa***	(105, 106),(307, 308),(403, 404)	$\frac{1}{4}$	$\frac{1}{2}$	$\frac{1}{4}$	3	4	5.25	1.75	3.86	0.049
***aa*, *aa***	(120, 121),(122, 123),(315, 316), (405, 406)	0	0	1	0	5	0	5	0	1
***A_*, *aa***	(111, 112), (201, 202)	0	$1-\frac{q}{2}$	$\frac{q}{2}$	4	0	4 − 2q	2q	1.37	0.24
***a_*, *aa***	(113, 114),(303, 304)	0	$\frac{1-q}{2}$	$\frac{1+q}{2}$	0	4	2 − 2q	2 + 2q	1.30	0.25
***Aa*, *A_***	(203, 204), (401, 402)	$\frac{2-q}{4}$	$\frac{1}{2}$	$\frac{q}{4}$	2	0	$2-\frac{q}{2}$	$\frac{q}{2}$	0.29	0.59
***A_*, *A_***	(407, 408)	$\frac{{\left(2-q\right)}^{2}}{4}$	$\frac{q(2-q)}{2}$	$\frac{q^{2}}{4}$	1	0	$1-\frac{q^{2}}{4}$	$\frac{q^{2}}{4}$	0.07	0.79
**Total Chi-square**									7.42	0.28

Geno = Genotypes; Offs = Offspring; Obs. No. = Observed number; Exp. No. = Expected number; BB: blue-black; WG: white-gold; *q* is the estimated probability of allele *a* in the population.

Moreover, we tested the hypothesis of inheritance mode ii which assumed that there was no genetic effect on this trait. In other words, the transmission was random or independent. Our results rejected the hypothesis with statistically significant *p* value, 4.45ⅹ10^−4^ (Table 3). After ascertainment correction by omitting the probands, the p-values obtained for the two transmission modes were 0.53 and 1.10ⅹ10^−3^, respectively (S1 and S2 Tables). These results highly suggested that our hypothesis of a major autosomal gene with a recessive trait allele controlled transmission was much more preferred than the hypothesis of independent transmission. Furthermore, we tested more transmission models using SAGE software. We started with a sporadic model assuming no familial or residual association in the data (AIC = 91.72, Table 4). However the sporadic model with FA was a better fitting one (AIC = 86.95) which suggested heritable effects or shared environment effects for the BB color perception. Based on this, we therefore assumed familial/residual associations in the remaining models. To investigate the effect from gender and age, we incorporated them as covariates in our models, but the inclusion of either decreased the fitting of model (data not shown). These covariates were subsequently dropped from all analyses reported in this study.

**Table 3:** Summary of the χ^2^ test in nuclear families under the hypothesis of independent transmission.

Parent Pheno.	Parent pairs	Obs. No.		Exp. No.		χ^2^	*P*-value
		WG	BB	WG	BB		
**WG, BB**	(103, 104), (107, 108), (111, 112), (201, 202), (305, 306), (311, 312), (313, 314)	7	7	9.52	4.48	2.08	0.15
**BB, BB**	(120, 121), (122, 123), (315, 316), (405, 406)	0	5	3.4	1.6	10.63	0.001
**WG, WG**	(105, 106), (203, 204), (307, 308), (401, 402), (403, 404), (407, 408)	6	4	6.8	3.2	0.29	0.59
**BB, missing**	(113, 114), (301, 302), (303, 304), (309, 310)	3	6	6.12	2.88	4.97	0.03
**Total Chi-square**						17.97	4.45×10^−4^

Pheno = Phenotype; Obs. No. = Observed number; Exp.No. = Expected number; WG: white-gold; BB: blue-black.

**Table 4:** Parameter estimates from segregation analysis of the dress color perception in four complex pedigrees.

Hypothesis		Susceptibilities			Genotype Frequencies			Transmission Probabilities			
	*q*_*a*_	*β*_*aa*_	*β*_*Aa*_	*β*_*AA*_	*P*_*aa*_	*P*_*Aa*_	*P*_*AA*_	*τ*_*aa*_	*τ*_*Aa*_	*τ*_*AA*_	AIC
**Sporadic**	-	0.15	0.15	0.15	-	-	-	-	-	-	91.72
**Sporadic with FA**	-	0.92	0.92	0.92	-	-	-	-	-	-	86.95
**Mendelian major gene**											
**Dominant**	0.67	0.84	**= *β***_***aa***_	10.97	0.44	0.45	0.11	[1]	[0.50]	[0]	88.42
**Recessive**	0.39	71.18	**= *β***_***AA***_	0.70	0.15	0.47	0.38	[1]	[0.50]	[0]	87.74
**Additive**	0.092	-146.73	-73.02	0.68	0.008	0.17	0.82	[1]	[0.50]	[0]	90.88
**Non-Mendelian major gene**											
**Dominant**	0.73	0.89	**= *β***_***aa***_	709.26	0.54	0.39	0.07	0.91	0.48	0.81	77.64
**Recessive**	0.29	709.23	**= *β***_***AA***_	0.88	0.08	0.41	0.51	1	0.09	0.33	**75.66**
**Additive**	0.96	0.89	352.78	704.68	0.93	0.07	0.001	0.96	0.99	0	**75.67**
**Environmental**	0.97	0.90	556.90	371.27	0.93	0.07	0.001	0.97	0.97	0.97	**75.68**
**tau AB free**	0.14	-215.41	-107.41	0.58	0.02	0.23	0.78	1	0.27	0	91.68

Among all transmission models, the non-Mendelian recessive model was the most parsimonious one with AIC = 75.66, which was consistent with our previous conclusion. Two other comparable models were non-Mendelian additive model and environmental model, with AIC values 75.67 and 75.68, respectively. For the most parsimonious non-Mendelian recessive model, the estimated allele frequency was relatively lower (*q*_a_ = 0.29) than the estimation from the Twitter poll. On the other hand, phenotypes apparently not carrying the BB trait allele can also have the probability of transmitting the BB trait (τ_AA_ = 0.33). The transmission mode of BB as a dominant one with complete penetrance was found to be possible but less likely than the other two non-Mendelian models (AIC = 77.64).

Altogether, these results provided evidence consistent with a major gene controlled segregation for the dress color perception. Our analyses also indicated that the segregation of BB trait might be in an environmental fashion or a gene-environment interaction fashion.

## Discussion

In summary, we have for the first time demonstrated that there was preliminary evidence of a genetic basis influencing the color perception of a blue-black dress from the photograph. We demonstrated that a major autosomal gene controlled transmission was more fitting than the other presumed modes with regard to “the dress” phenomenon. Our study also indicated the importance of environmental or epigenetic factors in this color perception trait. It should be noted that the conclusion we made should be understood in terms of the color perception from the dress photo, instead of the real colors of the dress. The phenotypes defined in our analyses should be the variation of color perception with specific type of lights as background. Importantly this preliminary study does not preclude the possibility of a purely environment influenced mode or polygenic mode of the color perception segregation.

Beside the genetic effects, other risk factors such as environment may contribute to this trait. Our finding of the possible environmental reasons influencing perception has been indicated in previous studies. In 2015, a consumer genetics company, 23andMe, published results of a genomic study on their blog, and implied that type of childhood residence was associated with the dress color perception trait. Although no significant association between genetic factors and the color perception trait was detected in that study, it underscored the complex interactions of genetics and environmental factors [9]. Moreover it revealed age to be a determinant on the dress color perception as older people were found to be more likely to see WG colors [10].

Another pedigree study analyzed data from 28 families and showed no clear trend for the perceptions of parents to predict those of their children [11]. Among the four families where both parents saw blue and black, four of 12 offspring saw white and gold. That report ostensibly contradicts our current report. Incomplete penetrance of the trait locus is a possible explanation of this discrepancy which warrant further and larger scale data collection and analysis.

As a preliminary study, this report is to draw our attention to the possibility that there may exist a genetic basis of the color perception, although the larger number of families and sizes are necessary in order to prove or exclude this possibility. A notable challenge in studying the color perception is the existence of switchers in the population. The switchers see the dress color differently from time to time [4]. We did not find evidence of color switch in our data, and hence considered the color perception as a dichotomous trait in the modeling. A latent variable model through an unobserved continuous variable can be considered in the presence of switchers in the data. These topics warrant further studies in the future.

Finally, to validate our preliminary finding and eventually locate the functional trait locus, further genomic and functional studies are warranted. The scope of those studies is far beyond the present report.

## Supporting Information

S1 Table Summary of χ^2^ test in nuclear families after ascertainment correction. This is under the hypothesis of a single dominant gene controlled transmission.
(DOCX)

S2 Table Summary of the χ^2^ test in nuclear families after ascertainment correction. This is under the hypothesis of independent transmission.
(PDF)
