# Supplementary material for: Segregation Analysis Suggests That a Genetic Reason May Contribute to “the Dress” Colour Perception
Source: PLoS One. 2016 Oct 21;11(10):e0165095. doi: 10.1371/journal.pone.0165095 (PMC5074562; doi:10.1371/journal.pone.0165095)
Supplement: S1 Table — This is under the hypothesis of a single dominant gene controlled transmission. (DOCX) [file pone.0165095.s001.docx]

**S1 Table. Summary of test in nuclear families after ascertainment correction.** This is under the hypothesis of a single dominant gene controlled transmission.

| Parent  Geno. | Parent pairs | $P{}_{AA}$  in Offs. | $P{}_{Aa}$  in Offs. | $P_{aa}$  in Off. | Obs. No. | | Exp. No. | |  | *P-* value |
| --- | --- | --- | --- | --- | --- | --- | --- | --- | --- | --- |
|  |  |  |  |  | **WG** | **BB** | **WG** | **BB** |  |  |
| *Aa, aa* | (103, 104),(107, 108),(301, 302),(305, 306),(309, 310),(311, 312),(313, 314) | 0 | $\frac{1}{2}$ | $\frac{1}{2}$ | 6 | 7 | 6.5 | 6.5 | 0.08 | 0.78 |
| *Aa, Aa* | (105, 106),(307, 308),(403, 404) | $\frac{1}{4}$ | $\frac{1}{2}$ | $\frac{1}{4}$ | 3 | 3 | 4.5 | 1.5 | 2.00 | 0.16 |
| *aa, aa* | (120, 121),(122, 123),(315, 316), (405, 406) | 0 | 0 | 1 | 0 | 5 | 0 | 5 | 0 | 1 |
| *A_, aa* | (111, 112), (201, 202) | 0 | $1-\frac{q}{2}$ | $\frac{q}{2}$ | 4 | 0 | $4-2q$ | $2q$ | 1.37 | 0.24 |
| *a_, aa* | (113, 114),(303, 304) | 0 | $\frac{1-q}{2}$ | $\frac{1+q}{2}$ | 0 | 4 | $2-2q$ | $2+2q$ | 1.30 | 0.25 |
| *Aa, A_* | (203, 204), (401, 402) | $\frac{2-q}{4}$ | $\frac{1}{2}$ | $\frac{q}{4}$ | 2 | 0 | $2-\frac{q}{2}$ | $\frac{q}{2}$ | 0.29 | 0.59 |
| *A_, A_* | (407, 408) |  | $\frac{q(2-q)}{2}$ |  | 1 | 0 |  |  | 0.07 | 0.79 |
| Total Chi-square | | | | | | | | | 5.11 | 0.53 |

Geno=Genotypes; Offs=Offspring; Obs. No.= Observed number; Exp. No.= Expected number;

BB: blue-black; WG: white-gold; *q* is the estimated probability of allele *a* in the population.
