# Supplementary material for: Segregation Analysis Suggests That a Genetic Reason May Contribute to “the Dress” Colour Perception
Source: PLoS One. 2016 Oct 21;11(10):e0165095. doi: 10.1371/journal.pone.0165095 (PMC5074562; doi:10.1371/journal.pone.0165095)
Supplement: S2 Table — This is under the hypothesis of independent transmission. (PDF) [file pone.0165095.s002.pdf]

S2 Table. Summary of the χ^2 test in nuclear families after ascertainment correction. This is under the hypothesis of independent transmission.

Parent Pheno.	Parent pairs	Obs. No.		Exp. No.		χ^2	*P*-value
		WG	BB	WG	BB		
**WG, BB**	(103, 104), (107, 108), (111, 112), (201, 202), (305, 306), (311, 312), (313, 314)	7	5	8.16	3.84	0.52	0.47
**BB, BB**	(120, 121), (122, 123), (315, 316), (405, 406)	0	5	3.4	1.6	10.63	0.001
**WG, WG**	(105, 106), (203, 204), (307, 308), (401, 402), (403, 404), (407, 408)	6	3	6.12	2.88	0.007	0.59
**BB, missing**	(113, 114), (301, 302), (303, 304), (309, 310)	3	6	6.12	2.88	4.97	0.03
**Total Chi-square**						16.12	1.10×10^{-3}

Pheno = Phenotype; Obs. No.= Observed number; Exp.No.= Expected number; WG: white-gold; BB: blue-black.
